# What can the global palliative care community learn from grassroots caregiving in the Philippines?

**DOI:** 10.1017/S1478951525100734

**Published:** 2025-09-08

**Authors:** Jose Eric Mella Lacsa

**Affiliations:** Theology and Religious Education, De La Salle University, Manila, Philippines

Dear Editor,

I am writing in response to the article titled “The caregiver’s journey: A qualitative study on the integration of family caregivers of advanced cancer patients in outpatient settings in Germany” by Petya Zyumbileva and colleagues, published in *Palliative & Supportive Care* (Zyumbileva et al. 2025). This study addresses a highly relevant issue in palliative care, highlighting that family caregivers in Germany’s outpatient cancer care system encounter considerable yet often overlooked challenges. The authors underscore the pressing need for improved integration and support of caregivers through individualized, proactive approaches, initiated at the time of diagnosis and encompassing early palliative involvement, routine needs assessments, and the adoption of digital solutions, to enhance the overall quality of cancer care.

This message resonates powerfully in the Philippine context, where cancer caregiving unfolds within families often strained by poverty, limited access to care, and cultural expectations of sacrifice. In many homes across the archipelago, caregiving is not delegated to professionals but taken up by mothers, daughters, spouses, often without training, respite, or recognition (Dumlao-Osório et al. 2025). A sari-sari store doubles as a pharmacy, a prayer group becomes a counseling circle, and public transport is repurposed into an emergency vehicle when hospitals are far and funds are scarce.

Unlike in high-resource settings, the integration of family caregivers into outpatient care in the Philippines cannot rely solely on existing health infrastructure. Instead, it demands creative, community-rooted solutions. Barangay health workers, faith-based organizations, and even local radio stations could play vital roles in identifying caregiver stress, providing basic psychosocial support, and disseminating accessible palliative care information (Corpuz 2023). In the absence of robust digital ecosystems, simple SMS-based systems could be deployed to check in on caregivers or remind them of medical appointments.

Yet, even amid these limitations, the Philippine experience offers lessons for the global community. Cultural values like *pakikipagkapwa* (shared humanity) and *bayanihan* (communal solidarity) are powerful assets that can be harnessed in designing grassroots caregiver support systems. Global south innovations, often born out of necessity, may serve as models for rehumanizing caregiving elsewhere (Solis 2023). Zyumbileva et al.’s study prompts a vital global conversation: How do we care for those who care? For countries like the Philippines, this question is urgent. Without supporting family caregivers, we risk building cancer care systems that are clinically efficient but morally hollow. A truly compassionate and globally relevant palliative care model must elevate caregivers, not as invisible extensions of the patient, but as individuals with distinct needs, voices, and rights to healing.
